# Comorbid Personality Disorders in Individuals With an At-Risk Mental State for Psychosis: A Meta-Analytic Review

**DOI:** 10.3389/fpsyt.2019.00429

**Published:** 2019-07-05

**Authors:** Tommaso Boldrini, Annalisa Tanzilli, Maria Pontillo, Antonio Chirumbolo, Stefano Vicari, Vittorio Lingiardi

**Affiliations:** ^1^Department of Dynamic and Clinic Psychology, Faculty of Medicine and Psychology, Sapienza University of Rome, Rome, Italy; ^2^Department of Developmental Psychology and Socialization, University of Padova, Padova, Italy; ^3^Child and Adolescence Neuropsychiatry Unit, Department of Neuroscience, Children Hospital Bambino Gesù, Rome, Italy; ^4^Department of Psychology, Faculty of Medicine and Psychology, Sapienza University of Rome, Rome, Italy

**Keywords:** personality disorders, ultra high risk (UHR), clinical high risk (CHR), high risk (HR), early detection and prevention

## Abstract

Increasing evidence shows that personality pathology is common among patients at clinical high risk (CHR) for psychosis. Despite the important impact that this comorbidity might have on presenting high-risk psychopathology, psychological functioning, and transition to full psychotic disorders, the relationship between personality syndromes and CHR state has received relatively little empirical attention. The present meta-analytic review aimed at 1) estimating the prevalence rates of personality disorders (PDs) in CHR individuals and 2) examining the potential role of PDs in predicting transition from CHR state to a full-blown psychotic disorder. The systematic search of the empirical literature identified 17 relevant studies, including a total of 1,868 CHR individuals. Three distinct meta-analyses were performed to provide prevalence estimates of PDs in the CHR population. The first and more comprehensive meta-analysis focused on any comorbid PD (at least one diagnosis), the second one focused on schizotypal personality disorder (SPD), and the last one focused on borderline personality disorder (BPD). Moreover, a narrative review was presented to define the predictive role of personality disorders in promoting more severe outcomes in CHR patients. The findings showed that the prevalence rate of personality disorders in CHR patients was 39.4% (95% CI [26.5%–52.3%]). More specifically, 13.4% (95% CI [8.2%–18.5%]) and 11.9% (95% CI [0.73%–16.6%]) of this clinical population presented with SPD and BPD, respectively. Finally, the studies examining the effects of baseline personality diagnoses on conversion to psychotic disorders showed contradictory and insufficient results concerning the potential significant impact of SPD. Conversely, no effect of BPD was found. This meta-analytic review indicated that the CHR population includes a large subgroup with serious personality pathology, that may present with attenuated psychotic symptoms conjointly with distinct and very heterogeneous personality features. These findings support the need for improved understanding of both core psychological characteristics of CHR patients and differentiating aspects of personality that could have relevant clinical implications in promoting individualized preventive interventions and enhancing treatment effectiveness.

## Introduction

Very early detection and intervention in the course of illness are considered the crucial goals for realizing meaningful improvements in the outcome of schizophrenia spectrum disorders. Much research and many clinical works over the last 20 years have explored the possibility of intervention before the onset of the full psychotic disorder, in order to preempt negative clinical outcomes. These efforts focused on the pre-psychotic or “prodromal” stages of illness, which have been defined as the period of time characterized by increasing changes in thinking, feeling, and behaving from a person’s premorbid mental state and level of functioning up to the appearance of psychotic features (1, 2). To promote early intervention, it is critical to prospectively assess the psychosis liability (i.e., detecting the true risk of developing a psychotic illness in specific help-seeking populations in an accurate manner).

Two sets of operational criteria for diagnosing the clinical high risk (CHR) state have been developed and tested: The Ultra-High Risk (UHR) and the Basic Symptom (BS) criteria. The UHR state has been operationalized by the presence of one or more of the following: 1) attenuated psychotic symptoms (APS), 2) brief limited intermittent psychotic symptoms (BLIPS), or 3) trait vulnerability plus a marked decline in psychosocial functioning (Genetic Risk and Deterioration Syndrome, GRD) [for a review, see Ref. (3)]. On the other hand, BSs have been conceptualized as the most immediate symptomatic expression of neurobiological aberrations, underlying the development of schizophrenia spectrum disorders (4). These symptoms could be described in terms of subjective subclinical disturbances in different domains (i.e., perception, thought processing, language, and attention) that are phenomenologically distinct from classical psychotic symptoms, by reason of their self-experienced nature and fully preserved insight and reality testing (5, 6).

Reliable and valid instruments have been developed and refined to identify the UHR (7, 8) and the BS groups (9). CHR subjects who met UHR or BS criteria or a combination of both showed a transition rate to a full-flagged psychotic disorder ranging from 18% after 6 months, 22% after 1 year, and 29% after 2 years to 36% after 3 years (10). Despite the promising predictive validity of these criteria, the rates of “false positives” and the most recent concerns of lower transition rates [for a deeper discussion, see Ref. (11)] have prompted researchers to identify additional clinical conditions and/or manifestations, in order to improve prediction and reduce the rate of converters.

It has been argued that premorbid personality disorders (PDs) may represent a noteworthy and relevant “vulnerability marker” or risk factor for psychotic disorders, especially within neurodevelopmental processes in adolescence and young adulthood (12). Due to the heterotypic continuity in mental disorders’ development, as well as putative shared genetic or early developmental etiological factors, emerging dysfunctional personality patterns might promote a range of severe clinical pictures and possibly end in first-episode schizophrenia or another full-blown psychotic disorder (13–16). More generally, the relationship between personality and psychotic disorders can be explained by at least three explanatory models (17). First, personality and psychopathology may have a *pathoplastic* relationship, whereby the former modifies the phenotypic expression of the latter—and conversely. Second, the putative presence of common etiological and genetic factors may hesitate in a *spectrum* relationship, whereby personality and psychotic disorders fail to act as distinct entities—as in the case of schizotypal personality disorder (SPD) and schizophrenia (18–19). And third, personality and psychotic disorders may have a *causal* (etiological and possibly bidirectional) relationship, whereby individual patterns of thinking, feeling, behaving, and relating to others hesitate or contribute to the onset of a mental disorder, just as a severe or chronic psychotic disorder can itself contribute to important changes in personality^1^. Considering the clinical heterogeneity of CHR populations (22), as well as the lack of prognostic specificity of attenuated psychotic symptoms (23), exploring personality pathology in CHR individuals may aid in elucidating the etiopathogenetic pathways contributing to the onset of psychotic disorders.

Moreover, irrespective of their relationships with psychosis, personality pathology represents a very important threat and negative factor for positive therapy outcomes, considering its predominant role in how patients respond to treatment. Thus, the need to focus on personality characteristics in CHR individuals seems apparent: carefully understanding the patients’ patterns of thinking, feeling, coping, interpersonal functioning, experiencing of self and others, in which mental health problems are rooted, can be very useful for making more accurate diagnostic formulations, as well as for providing a road map for the implementation of preventive treatment strategies and intervention programs in this specific population.

Nevertheless, to the best of our knowledge, no meta-analytic review of empirical studies on comorbid personality syndromes in CHR individuals was conducted. The present study aimed at 1) estimating the prevalence rates of PDs in individuals at CHR of first-episode psychosis and 2) examining the potential role of personality pathology in predicting transition to full-flagged psychotic disorders.

## Methods

The main research hypothesis and the study protocol were decided *a priori*. The present meta-analytic review was conducted in accordance with the Preferred Reporting Items for Systematic Reviews and Meta-Analysis (PRISMA) guidelines (24).

### Search Strategy

We performed a multi-step literature search using the following keywords: (high AND risk [MeSH Terms] AND psychotic disorders [MeSH Terms] OR psychosis OR risk [MeSH Terms] AND psychotic disorders [MeSH Terms] OR psychosis OR early diagnosis [MeSH Terms] AND psychotic disorders [MeSH Terms] OR psychosis OR prodrom* AND psychotic disorders [MeSH Terms] OR psychosis) AND (personality [MeSH Terms] OR personality disorders [MeSH Terms]).

First, we conducted a systematic literature search in MEDLINE, PubMed, Scopus, Web of Science, and PsychINFO databases, including all the articles published until September 2018, in the English language. Second, the reference lists of the articles included in the review were manually checked for any studies not identified by the computerized literature search. The abstracts from the articles identified through this process were then screened, and the full texts were retrieved for further examination in relation to the inclusion and exclusion criteria (as detailed below). The database search, study selection, and data extraction were carried out by two authors (the first and the second) independently. Disagreements were solved through consensus discussions among all the authors.

### Eligibility Criteria

Studies were considered eligible for inclusion in this review when they fulfilled the following criteria: 1) published as an original paper in a peer-reviewed journal; 2) involved CHR individuals as defined according to established international criteria and by validated assessments [e.g., Comprehensive Assessment of At Risk Mental State (CAARMS) (8); Structured Interview for Psychosis-Risk Syndrome (SIPS) (25)]; 3) evaluated comorbid PDs at baseline and/or reported the proportion of personality pathology in high-risk subjects with longitudinal transition to psychosis; and 4) evaluated PDs with reliable and validated instruments [e.g., Structured Clinical Interview for Diagnostic and Statistical Manual of Mental Disorders 4th ed. (DSM–IV) (26) Axis II Personality Disorders (SCID-II) (27)] . When two or more studies were from the same center, we contacted the authors to determine whether overlap existed in the respective samples; overlapping samples were excluded. When the proportion of comorbid personality diagnoses was not indicated in a retrieved article, we contacted the corresponding author to collect the additional data. Finally, when a study conveyed insufficient information to determine whether the selection criteria had been met, it was excluded from the review.

### Recorded Variables

The variables for each article included in the meta-analytic review were year of publication, sex and mean age of participants, inclusion criteria for the CHR state, psychometric instruments used to assess the psychosis risk, psychometric instruments used to assess PDs, prevalence rates of PDs in CHR individuals, duration of follow-up, criteria used to define transition to psychosis, and transition risk at different time points (%).

### Quality Assessment

To conduct the quality assessment of the studies included in this meta-analytic review, we adapted the Newcastle–Ottawa Scale (NOS) that has been adopted in recent meta-analyses [e.g., Ref. (28)]. This scale allows us to allocate a maximum of nine stars for the highest quality. Each study was independently assessed by the first and second authors to ensure interrater reliability. All authors double-checked and resolved inconsistency and disagreements on quality scoring.

### Statistical Analysis

The meta-analysis was performed using Comprehensive Meta-Analysis (CMA) software version 2 (Biostat, Inc) (29). CMA software allows for the meta-analysis of proportions using the number of events and the total sample. The effect sizes were weighted according to the inverse of their variances and their calculation was based on a random-effects model (30, 31). The effect size represented the proportion of current PD (at least one diagnosis), SPD, and borderline personality disorder (BPD) in subjects with a baseline high-risk state for psychosis. It has not been possible to measure other proportions because the number of studies that had evaluated PDs other than SPD and BPD at baseline was too small for a meta-analysis (<4).

## Results

### Retrieved Studies

**Figure 1:**
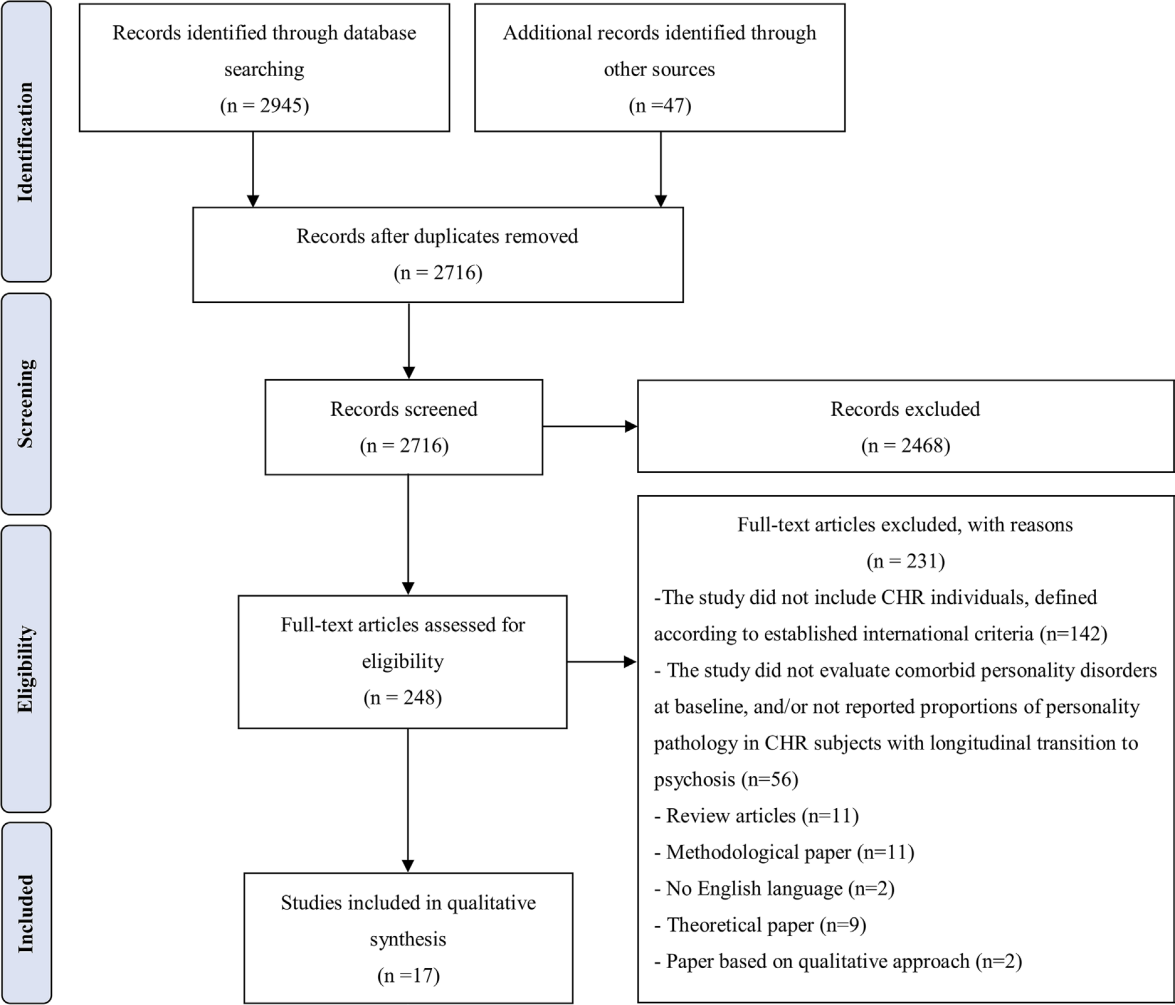
Preferred Reporting Items for Systematic Reviews and Meta-Analysis (PRISMA) Flowchart [see Ref. (20)].

The identification, selection, screening, and inclusion or exclusion of studies is extensively described in the flow chart (see **Figure 1**), in which reasons for article rejection are clearly indicated. The initial database search produced 2,945 records, and an additional 47 records were identified through the other sources previously described. After duplicates were removed, the first and second authors independently screened all titles and abstracts from the initial search to individuate the studies that were eligible for full‐text retrieval. We excluded 2,468 records because they did not meet the inclusion criteria, with an interrater agreement of 89%. The remaining 248 articles were retrieved for full‐text screening, and 231 were excluded for not meeting the inclusion criteria, with an interrater agreement of 84%. Uncertainties relating to an article’s final inclusion in the review (*n* = 23) were resolved by the independent judgment of the other authors.

Seventeen studies were included in the final review and then qualitatively and meta-analytically synthesized.

### Study Characteristics

**Table 1** shows the descriptive characteristics of the 17 included studies. All studies were published in English between 2001 and 2018, with CHR sample sizes ranging from 21 to 377 (M = 117.56; SD = 95.99; Mdn = 99.50). In summary, there were two main forms of diagnostic criteria used to define CHR features in help-seeking patients, the UHR and BS. The UHR state was independently assessed with the CAARMS (8) and the SIPS (7). In most of the studies included (*K* = 15), PDs were assessed administering clinical interviews based on DSM-IV diagnostic criteria [e.g., Structured Clinical Interview for DSM-IV (SCID-II), Structured Interview for DSM-IV Personality (SIDP-IV) (51), or Diagnostic Interview for DSM-IV Personality Disorders (DIPD-IV) (52)]. Otherwise, self-report measures were administered (*K* = 2). These instruments for assessing personality are based on a set of dimensional traits, and a PD diagnosis is assigned when one or more traits are clinically relevant (in other words, the scores obtained on specific scales must be greater than certain threshold values or cut-off). The cross-sectional design was the most commonly adopted (*K* = 7). In the studies where cross-sectional design was used, CHR subjects were compared with healthy volunteers (*K* = 1; 45), patients from a clinical population without a high-risk for psychosis (*K* = 4; 36, 39, 41, 46), healty volunteers and first-episode psychosis patients (*K* = 1; 48), or were assessed in terms of sociodemographic and clinical characteristics (*K* = 1; 38). Of the two case–control studies, the groups were CHR-treated patients who subsequently transitioned to full-threshold psychotic disorder (converters), and “controls” were patients who did not meet criteria for psychotic disorder in a follow-up period—ranging from 12 to 24 months. Of the studies that used a longitudinal design (*K* = 7), the follow-up length ranged from 6 months to 9.6 years. Psychosis transition was defined according to “standard” criteria [from the two major psychiatric diagnostic guidelines, DSM and International Classification of Diseases (ICD)] or criteria from the main UHR clinical assessment instruments (53).

**Table 1:** Study characteristics.

Study	Research center	HR sample	HR definition	Personality assessment instrument	Personality variable	Study Design	Notes
Bechdolf et al. (32)	9 early detection and intervention centres, Germany	*N* = 156 F = 50, M = 106 Age M = 23.86 years (SD = 4.89)	SIPS; SPI-A	Structured Clinical Interview for DSM-IV (SCID-II)	DSM-IV personality disorders	Longitudinal randomized controlled trial (RCT)	
Cannon et al. (33)	NAPLS	*N* = 364 F = 124, M = 240 Age M = 18.3 years (SD = 9.75	SIPS	SIPS defined schizotypal personality disorder (presence of only at least one year required)	Schizotypal personality disorder	Longitudinal	Same sample of Woods et al. (34)
Falkenberg et al. (35)	OASIS, UK	*N* = 221 F = 104, M = 117 Age M = 22.6 years (SD = 4.7)	CAARMS; SPI-A	Structured Clinical Interview for DSM-IV (SCID-II)	DSM-IV personality disorders	Longitudinal	
Gerstenberg et al. (36)	Switzerland	*N* = 21 F = 11, M = 10 Age M = 15.00 years (SD = 1.4)	SIPS	Structured Interview for DSM-IV Personality (SIDP-IV)	DSM-IV personality disorders	Cross-sectional	Psychiatrically hospitalized adolescents with nonpsychotic disorders
Klosterkötter et al. (37)	CER, Germany	*N* = 110 F = 51, M = 59 Age M = 28.8 years (SD = 9.75)	BSABS	PSE9	DSM-III personality disorders	Longitudinal	
Kotlicka-Antczak et al. (38)	Center clinical hospital of Lodz, Poland	*N* = 99 F = 54, M = 45 Age M = 19 years (SD = 3.56)	CAARMS	Structured Clinical Interview for DSM-IV (SCID-II)	DSM-IV personality disorders	Cross-sectional	
Lee et al. (39)	Clinic FORYOU, Korea	*N* = 63 F = 25, M = 38 Age M = 19.7 years (SD = 3.5)	SIPS	Structured Clinical Interview for DSM-IV (SCID-II)	Schizotypal personality disorder	Cross-sectional	
Lencz et al. (40)	RAP, New York	*N* = 42 F = 17, M = 25 Age M = 16.4 years (SD = 2.3)	SIPS	Structured Interview for DSM-IV Personality (SIDP-IV)	DSM-IV personality disorders	Cross-sectional	
Lim et al. (41)	Seoul Youth Clinic, Korea	*N* = 129 F = NR, M = NR Age M = 20.74 years (SD = 3.2)	SIPS	Structured Clinical Interview for DSM-IV (SCID-II)	DSM-IV personality disorders	Longitudinal	
Rosen et al. (42)	PRIME, USA	*N* = 29 F = 15, M = 14 Age M = 18.4 years (SD = 4.8)	SIPS	Diagnostic Interview for DSM-IV Personality Disorders (DIPD-IV)	DSM-IV personality disorders	Cross-sectional	
Ruhrmann et al. (43)	EPOS project, Europe	*N* = 245 F = 108, M = 137 Age M = 23.0 years (SD = 5.2)	SIPS; BSABS-P	SIPS defined schizotypal personality disorder (presence of only at least one year required)	Schizotypal personality disorder	Longitudinal	
Ryan et al. (44)	PACE, Australia	*N* = 131 F = 83, M = 48 Age M = range from 15 to 24	CAARMS	Structured Clinical Interview for DSM-IV (SCID-II)	Borderline personality disorder	Longitudinal	
Schultze-Lutter et al. (45)	Cologne early detection and intervention service, FETZ, Germany	*N* = 100 F = 24, M = 76 Age M = 24 years (SD = 6)	SPI-A	Self-report version of the Aachener Merkmalsliste für Persönlichkeitsstörungen (SAMPS)	Personality traits and disorders	Case control study (converters vs. non-converters)	
Sevilla-Llewellyn-Jones et al. (46)	CAMEO Early Intervention in Psychosis Service, UK	*N* = 40 F = 21, M = 19 Age M = 21.65 years (SD = 2.64)	CAARMS	Millon Multiaxial Inventory, version III (MCMI-III)	Personality traits	Cross-sectional	
Spada et al. (47)	Italy	*N* = 22 F = 10, M = 12 Age M = 16.1 years (SD = 1.02)	CAARMS	Structured Clinical Interview for DSM-IV (SCID-II)	DSM-IV personality disorders	Cross‐sectional	
Thompson et al. (48)	PACE, Australia	*N* = 96 F = 52, M = 44 Age M = 18.3 years (SD = 2.7)	CAARMS	Structured Clinical Interview for DSM-IV (SCID-II)	Borderline personality disorder	Case–control study	
Woods et al. (34)	NAPLS, USA	*N* = 377 F = 143, M = 234 Age M = 18.2 years (SD = NR)	SIPS	Structured Interview for DSM-IV Personality Disorders, Diagnostic Interview for DSM-IV Personality Disorders, or SCID-IV Axis II personality Disorders	DSM-IV personality disorders	Case–control study (converters vs non-converters)	

SIPS, Structured Interview for Prodromal Symptoms; CAARMS, comprehensive assessment of at-risk mental states; BSABS, Bonn Scale for the Assessment of Basic Symptoms; BSABS-P, Bonn Scale for the Assessment of Basic Symptoms: Prediction List (49); SPI-A, Schizophrenia Proneness Instrument-Adult Version; SPI-CY, Schizophrenia. Proneness Instrument Child-Youth; NAPLS, North American Prodromal Longitudinal Study; PACE, Personal Assessment and Crisis Evaluation Clinic; EPOS, European Prediction of Psychosis Study; RAP, Zucker Hillside Recognition and Prevention Program; CER, Cologne Early Recognition; PRIME, Prevention through Risk Identification; PSE9, Present State Examination, Ninth Version (50).

### Overall Quality Assessment

The quality assessment showed good interrater agreement (81.5%), with nine studies receiving high quality scores (≥8 NOS stars) and others receiving medium evaluation (5 ≤ NOS stars ≤ 7). A table explaining the calculation of the quality score for each study is available in **Supplementary Material**. Seven authors were contacted in order to clarify information relating to the quality criteria: one replied with relevant information, two did not reply, and in the remaining four cases, the email bounced back.

### Study Findings

#### Personality Disorders in CHR Individuals

Seventeen empirical investigations meeting the inclusion criteria of the present study were considered to evaluate the prevalence rate of PDs in individuals at CHR for psychosis. Personality pathology was mostly assessed according to the DSM-IV Axis II diagnostic category criteria (26). Three meta-analyses focused on the prevalence of PDs (at least one diagnosis) (Meta-Analytic Results on Prevalence Rate of Any Personality Disorder), SPD (Meta-Analytic Results on Prevalence Rate of SPD), and BPD (Meta-Analytic Results on Prevalence Rate of BPD), respectively, in subjects with a baseline high risk state for psychosis.

It is noteworthy that some studies included in these meta-analyses (*K* = 6) (32, 34, 37, 40, 42, 45) also reported data for other distinct concurrent personality syndromes. However, the paucity and heterogeneity of such empirical data did not allow us to perform additional meta-analytic estimations. In general, paranoid, schizoid, antisocial, and avoidant PDs were the most common syndromes, with prevalence rates ranging from 6% to 12%, 3% to 12%, 1% to 14%, and 10% to 26%, respectively. Conversely, the prevalence rates of histrionic, narcissistic, obsessive-compulsive, and dependent PDs were weaker (less than 5%).

Moreover, some studies (*K* = 2) (45, 46) used self-report instruments to assess PDs, whereas other studies employed clinical interviews (*K* = 15). It was not possible to compare these studies and to evaluate the influence of PD assessment method as a potential moderator variable due to the limited number of empirical investigations based on self-report evaluation. However, the results seem to indicate a potential impact of assessment method on the prevalence rate of PDs in all meta-analytical estimations [see, in particular, Ref. (46)].

##### Meta-Analytic Results on Prevalence Rate of Any Personality Disorder

**Figure 2:**
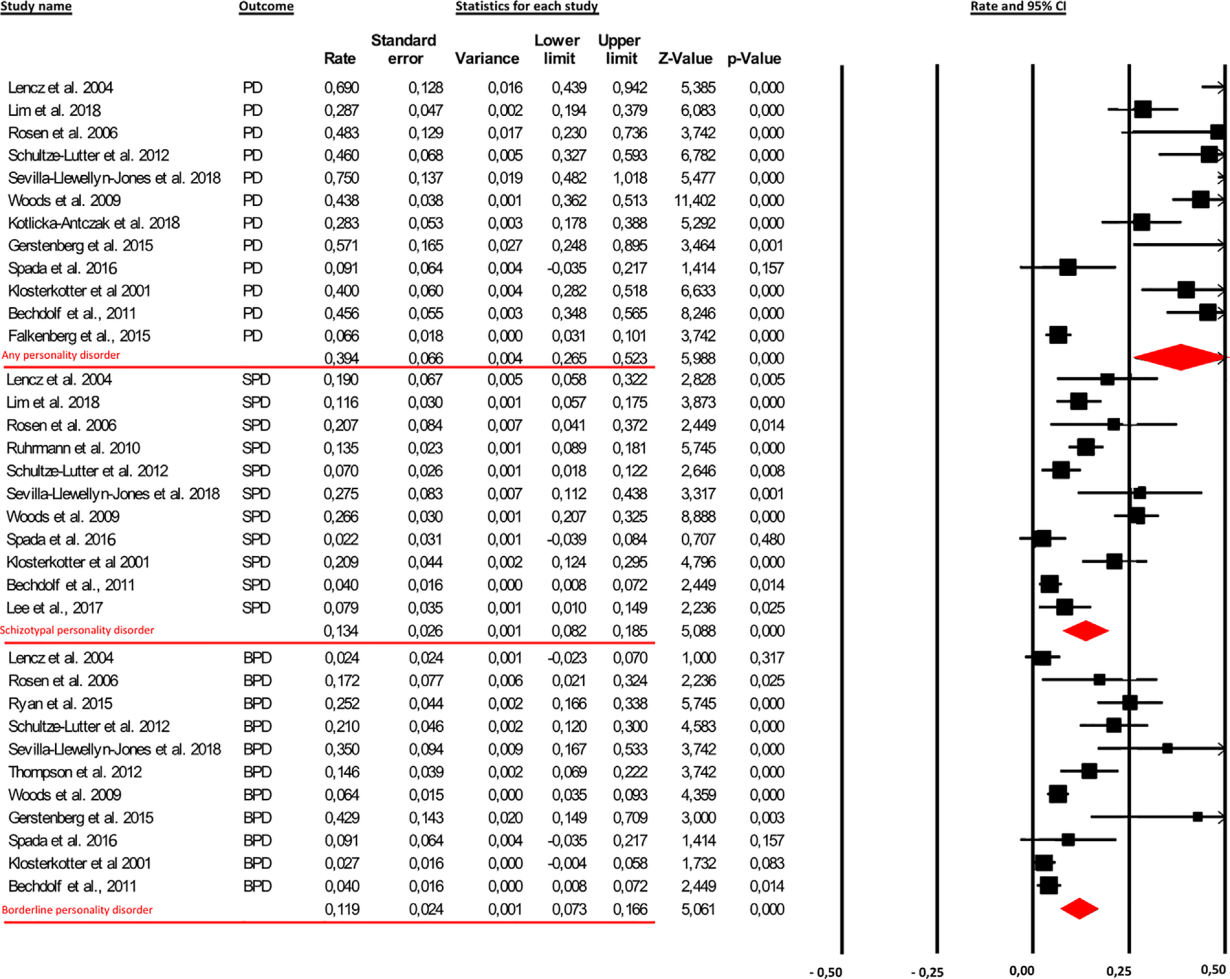
The findings showed that the prevalence rate of comorbid personality diagnoses in clinical-high-risk (CHR) patients was 39.4% [95% Cl (26.5%–52.3%)]. More specifically, 13.4% [95% Cl (8.2%–18.5%)] and 11.9% [95% Cl (0.73%–16.6%)] of this clinical population presented with the schizotypal personality disorder (SPD) and borderline personality disorder (BPD), respectively.

From our database, 12 samples were included in the first meta-analytical estimate, relating to a total of 1,346 CHR subjects [male 53.3%; mean age 20.36 (SD = 3.93)]. These subjects were assessed at baseline for any PDs. All studies included in this meta-analytical estimation reported prevalence data for all PDs. The meta-analysis found that comorbid baseline PDs (at least one diagnosis) were present in 39.4% of high-risk subjects (95% CI [26.5%–52.3%]; **Figure 2**).

##### Meta-Analytic Results on Prevalence Rate of SPD

Eleven samples were included in the second meta-analytical estimate, relating to a total of 1,313 CHR subjects [male 54.84%; mean age 20.95 (SD = 3.71)]. These subjects were assessed at baseline for SPD. The first and second meta-analysis differ in four studies: two (39, 43); reported data of SPD but did not specify prevalence rates of other PDs, whereas the other two (36, 38) provided data for other PDs without clarifying the prevalence rate for SPD. Moreover, one study (33) was excluded because it reported data from the same sample as Woods and colleagues (Woods and colleagues 2009). The results showed that comorbid SPD was present in 13.4% of high-risk subjects (95% CI [8.2%–18.5%]; **Figure 2**).

##### Meta-Analytic Results on Prevalence Rate of BPD

Eleven samples were included in the third meta-analytical estimate, relating to a total of 1,124 CHR subjects [male 57.6%; mean age 20.03 (SD = 4.30)]. These subjects were assessed at baseline for BPD. The first and third meta-analysis differ in five studies: two of them (44, 48) provided data of BPD but did not specify prevalence rates of other PDs, whereas the other three (38, 41, 44) reported data for other PDs without clarifying the prevalence rate for BPD. Comorbid BPD was present in 11.9% of high-risk subjects (95% CI [0.73%–16.6%]; **Figure 2**).

### Personality Disorders as Potential Predictors of Transition to Psychosis

Eight studies included in this systematic review were considered, in order to examine the impact of comorbid personality pathology on transition to full-flagged psychotic disorders (see **Table 2**). Overall, taking into account only the longitudinal studies (*K* = 6) and excluding case-control ones (*K* = 2), it is important to note that 341 of a total of 1,019 UHR subjects developed a psychotic episode (33.4%).

**Table 2:** Study findings on the impact of comorbid personality disorders (PDs) on transition to psychosis.

Study	Study design	Follow-up	Outcome measure(s)/transition	Personality assessment instrument	Rates of transition%	Predictor analyses	Main findings
Cannon et al. (33)	Longitudinal	2.5 years of follow-up	Transition to psychosis was assessed by SIPS.	SIPS defined schizotypal personality disorder (presence of only at least 1 year required)	35%	Kaplan–Meier survival analysis and Cox proportional hazard models.	SPDdid not predict conversion to psychotic disorders.
Klosterkötter et al. (37)	Longitudinal	9.6 years of follow-up	Psychosis diagnoses was rated according to DSM-IV criteria.	PSE9	49.4% (N = 160)	Logistic analyses	Irrespective of the presence of CHR criteria, only schizotypal personality disorder of all baseline diagnoses was significantly related to the subsequent development of schizophrenia (*n* = 79) in the total sample.
Lim et al. (41)	Longitudinal	8 years of follow-up divided in two groups (a group from 2005 to 2009 and a group from 2009 to 2013)	Transition to psychosis was defined as having psychotic level symptoms based on the SIPS for more than 4 days per week	Structured Clinical Interview for DSM-IV (SCID-II)	In the 2005–2009 group, the transition rates at 2 and 3 years were 25.3% and 31.1%, respectively. In the 2009–2013 group, the transition rates at 2 and 3 years were 4.4% and 25.7%, respectively.	Kaplan–Meier survival analysis and Cox proportional hazard models	Early referral and axis II comorbidities other than SPD were associated with the declining transition rate.
Ruhrmann et al. (43)	Longitudinal	18 months of follow-up	Transition to psychosis was assessed by SIPS. The diagnostic category of transition was determined by applying *DSM-IV* criteria for psychotic disorders and affective disorders with psychotic features.	SIPS defined SPD (presence of only at least one year required)	19%	Kaplan–Meier survival analysis and Cox proportional hazard models	SIPS-defined schizotypal personality disorder was one of six predictors of psychosis included in the predictor model
Ryan et al. (44)	Longitudinal	6–12 months of treatment.	Transition to psychosis was assessed by applying DSM-IV-TR criteria for psychotic disorders.	Structured Clinical Interview for DSM-IV (SCID-II)	13.9%	Direct logistic regression analysis	A quarter (25.2%) of UHR patients (*N* = 180) present with concurrent borderline personality features.
Schultze-Lutter et al. (45)	Case–control study [converters (*N* = 50) vs. non-converters (N = 50)]	1 year follow-up	Transition to psychosis in non-converters sample was assessed by applying DSM-IV criteria for psychotic disorders.	Self-report version of the Aachener Merkmalsliste für Persönlichkeitsstörungen (SAMPS)	/	Stepwise binary logistic regression analyses (no longitudinal) case-control (converters vs non-converters)	Unexpectedly, SPD was infrequent and did not predict conversion. Only schizoid subscale score was a significant though weak predictor of conversion; in particular items “lack of close friends or confidants other than first-degree relatives” and “emotional detachment observed by others”.
Sevilla-Llewellyn-Jones et al. (45)	Longitudinal	3 years of follow-up	The severity of psychotic symptoms was assessed by Positive and Negative Syndromes Scale (PANSS) (54)	Millon Multiaxial Inventory, version III (MCMI-III)	5%	Logistic regression analyses	The low transition rate observed in the sample precluded the possibility of testing the predictive power of maladaptive personality traits.
Thompson et al., (47)	Case–control study [converters (*n* = 48) vs non-converters (n = 48)]	24 months of follow-up	Psychosis diagnosis following transition was rated from the clinical files using the operational criteria in studies of psychotic illness (OPCRIT) computer algorithm.	Structured Clinical Interview for DSM-IV (SCID-II)	/	A combination of parametric and non-parametric analyses of variance	Co-occurring borderline personality disorder or borderline features does not appear to strongly influence the risk of short-term transition to psychosis or the risk of developing a non-affective psychotic disorder in UHR population.

SIPS, Structured Interview for Prodromal Symptoms; PSE9, Present State Examination, Ninth Version.

Two studies have investigated the presence of baseline comorbid PDs in predicting conversion to psychosis. Schultze-Lutter and colleagues (45) found that only schizoid features—in particular the “lack of close friends or confidants other than first-degree relatives” and “emotional detachment observed by others”—are able to significantly influence the subsequent development of psychosis despite the magnitude of this effect being quite weak. Contrary to their expectations, SPD was infrequent in CHR patients and did not predict conversion. Sevilla-Llewellyn Jones and colleagues (45) also examined the relationship between clinically significant personality traits and transitions to first-episode psychosis; however, the low transition rate in their sample precluded the possibility of testing the predictive power of overall personality traits.

Five studies based on different methodologies have longitudinally examined the role of SPD in developing a first episode of psychosis and provided inconsistent and mixed results. For example, SPD was the sole personality diagnosis related to conversion in the Cologne Early Recognition study (37). Moreover, schizotypal personality syndrome as defined by SIPS—that is, requiring a minimum presence of one year without changes in symptom severity—was one of six significant predictors of psychosis in the European Prediction of Psychosis Study (EPOS) (43). On the contrary, there was no evidence for a potential predictive effect of SPD in the North American Prodrome Longitudinal Study (NAPLS) (33). Notably, important differences between these studies can be traced, especially with regards to follow-up lengths and/or the mean age of samples. In particular, a significant psychosis-predictive role of SPD was found in samples with a greater mean age (e.g., 23 years) (43) and a longer follow-up period (e.g., 10 years) (37), suggesting that SPD can be considered as a distal trait risk factor that more significantly exerts its influence in the longer-term prognosis of CHR patients. Nevertheless, these inconclusive results do not allow us to establish whether the presence of SPD represents a more powerful predictor of transition to full psychotic disorder.

Three studies examined the potential predictive value of BPD for transition to psychosis in CHR sample. Schultze-Lutter and colleagues (45) and Ryan and colleagues (44) found that BPD did not predict the onset of psychotic disorder in CHR individuals. Moreover, Ryan and colleagues compared three groups of patients: “UHR only,” “UHR and likely borderline personality pathology,” and “UHR and borderline personality pathology,” showing no differences in the level of unusual thought content, non-bizarre ideas, perceptual abnormalities, or disorganized speech. These results seem to suggest that borderline personality features in CHR patients did not influence the clinical expression of attenuated psychotic symptoms; however, this lack of significant effect could also reflect an important limitation in the study related to potential biases in personality assessment procedures. In fact, borderline pathology was evaluated using a screening tool and employing self-report measures that may be problematic in the context of personality assessment [e.g., Refs. (55, 56)]. One additional study assessed borderline features administering a clinical interview and showed no statistically significant difference in the rate of transition to psychotic disorder in CHR patients with and without baseline full-threshold BPD (48).

Interestingly, baseline borderline pathology was not related to the onset of any particular type of psychotic disorder in the follow-up, rejecting the hypothesis that UHR patients with BPD features would be more likely to develop nonschizophrenia spectrum diagnoses or briefer psychotic episodes, which would be reflected in diagnoses, such as psychosis not otherwise specified (NOS) and brief reactive psychosis. Overall, despite several limitations [e.g. the use of self-report instruments (44, 45) and the small sample size (48)], the results from these three studies suggest that BPD does not increase the risk of transition and does not have a pathoplastic effect, neither with respect to the current clinical presentation nor with respect to the prognosis in CHR samples. Nevertheless, due to the paucity of studies on this topic, caution is required in drawing conclusions.

## Discussion

This is the first meta-analytic review focused on personality syndromes in patients at-risk for psychosis. Notably, this study sought to answer some specific questions: a) Is comorbid personality pathology prevalent among CHR individuals? b) Are some specific PDs more common than others? c) Is the risk of conversion to psychosis greater in CHR populations with comorbid PDs? Adopting strict inclusion criteria (specifically using appropriate and internationally shared definitions of UHR, as well as valid and reliable instruments for their detection), a total of 17 studies with 1,828 patients were included in this meta-analytic review (see **Table 1**).

Previous reviews and meta-analyses pointed out the huge variability of mental disorders in CHR individuals and high prevalence rates for many psychopathological syndromes or conditions [e.g., Ref. (57)]. In particular, comorbid depression and anxiety disorders have been identified as frequently marking the onset of the initial prodromes of psychosis (3). Conversely, the empirical literature regarding PDs and at-risk mental states is still limited and is not exhaustive. Despite the paucity and heterogeneity of existing research, this meta-analytic review has attempted to increase knowledge in the field. Specifically, the first aim of the study was to provide the prevalence rates of personality syndromes in the CHR population by performing three meta-analytic estimations. Second, the study aimed at exploring the potential impact of personality pathology in transition to psychosis.

### Prevalence Rate of PDs in CHR Individuals

Overall, the results showed that the prevalence of PDs is surprisingly high, with a baseline comorbidity present in 39.4% of CHR individuals. These data indicated that the CHR population includes a large subgroup with serious personality pathology, and 13.4% and 11.9% of CHR patients have comorbid SPD and BPD, respectively (**Figure 2**). These prevalence rates in CHR individuals are four times greater than those in the general population (58) and, for the most part, equivalent or superior to rates estimated in previous meta-analyses on other concurrent comorbid diagnoses (e.g., 40.7% for depressive disorders and 15.3% for anxiety disorders) (3).

### Prevalence Rate of SPD in CHR Individuals

The results of the second meta-analysis showed that SPD is common in high-risk patients. It is not surprising, as schizotypy is considered to be an indicator of being prone to psychosis and, therefore, a precursor to schizophrenia-spectrum disorders (19). Moreover, the widely used UHR criteria partially refer to the positive symptoms of schizotypy and SPD, such as unusual thought contents or magical thinking. However, it is necessary to clarify that SPD and CHR represent two specific and clearly delineated syndromes: While SPD is an enduring and persistent personality pattern, that requires signs and symptoms in at least five out of nine areas of psychological functioning and may sometimes precipitate the development of psychotic symptoms in a gradual manner, CHR conditions do not present stability during the past, meet fewer SPD symptoms, and show a dramatic progression of psychotic diseases (19). Clear delineation of the two syndromes also allows them to co-occur. For example, in Woods and colleagues’ (34) sample, 26% of prodromal patients met SPD criteria, whereas 67% of patients with an SPD diagnosis met prodrome criteria.

From a clinical standpoint, our results suggest the relevance of specific aspects of psychological functioning in CHR individuals with comorbid SPD diagnosis. These patients not only present with positive symptoms of schizotypy but also present with severe impairments in various personality domains. Beyond eccentric and idiosyncratic reasoning processes or unconventional beliefs, as well as perceptual distortions and an overall oddity in behavior and appearance, schizotypal patients show severe relational deficits marked by acute discomfort and reduced capacity for close relationships, affective flattening, and mental functioning impairment, characterized by difficulties in mentalizing processes and maladaptive metacognitions [e.g., Refs. (19, 59)]. These psychological characteristics may require the specific clinical attention of mental health professionals, as the treatment goal for CHR individuals should not be just preventing conversion to psychosis but also ameliorating the wider range of problems that members of this clinical population currently present (60).

### Prevalence Rate of BPD in CHR Individuals

The results of our last meta-analytic estimation revealed the association between BPD and at-risk mental states. Some studies included in this meta-analysis were specifically focused on BPD, also due to the historically complex diagnostic boundaries between borderline pathology and psychosis (61, 62).

Overall, some considerations regarding the high prevalence of BPD in CHR patients need to be addressed. First, BPD is typically associated with psychosis-like symptoms, such as transient paranoid ideation or severe dissociation (63). These symptoms are often trauma- and stress-related, unlikely predictive of a subsequent psychotic disorder (64) and differ from shizophrenia symptoms from a phenomenological standpoint (65). As a result, several borderline patients presenting with transient- and stress-related psychotic symptoms might be diagnosed as being at high risk for developing psychosis, generating false positives. Improving clinicians’ ability to distinguish between these different groups of patients would be meaningful and very useful for promoting clear case formulations and patient-tailored treatments [e.g., Ref. (48)].

Second, the comorbidity between BPD and CHR conditions could be influenced by other clinical variables. Substance abuse, for instance, is a recurrent clinical complication of borderline patients and is an important risk factor for the development of psychotic symptoms and disorders (66). Finally, the influence of putative, shared etiological factors between BPD and schizophrenia liability is notable. In particular, childhood traumatic experiences have been empirically associated with borderline pathology [e.g., Ref. (67)] and CHR status [e.g., Ref. (68)]. Emotional dysregulation and increased sensitivity to stress may be considered an endophenotype of psychosis, reflecting underlying gene–environment interactions associated with the impact of early trauma and stressful life events in vulnerable individuals (69). Consistent with this perspective, attenuated psychotic symptoms in CHR states could reflect core emotional dysregulation processes that would also account for their high comorbidity with anxiety and depressive diagnoses [see Refs. (3, 70, 71)]. In line with this possible explanation, it is important to highlight that borderline patients show, in general, severe emotional instability and are consequently vulnerable to experiencing overwhelming effects, including intense depression and anxiety. Considering all these relevant issues, the findings support potential interactions among emotional dysregulation, negative affectivity, and specific vulnerability for psychosis [e.g., Refs. (71–74)]. Further research is required to better clarify the complex processes underlying these associations.

### Impact of PDs in Transition to Psychosis

The second aim of this study was to investigate the predictive role of personality syndromes in the onset of psychotic disorders. The lack of clear evidence did not allow us to define specific disorders that are systematically associated with transition to full-blow psychotic disorders. The studies included in this review revealed contradictory and non-exhaustive findings about the potential significant impact of SPD, as well as no meaningful effect of BPD (see **Table 2**). However, how global characteristics of schizotypal personality are related to conversion to psychosis in high-risk individuals remains unclear. A possible explanation for these mixed results might be attributable to the different follow-up lengths and/or the mean age of different samples. From a clinical standpoint, attenuated psychotic symptoms might appear as a clinical manifestation or an exacerbation of schizotypy features, such as abnormal perceptual experiences, unusual beliefs, and transient quasi-psychotic episodes with intense illusions, auditory or other hallucinations, and delusion-like ideas (19). This perspective seems consistent with the current dimensional approach of schizophrenia-spectrum disorders (63), which assumes a distribution of schizotypal characteristics in the general population ranging from the adaptive and normal expression of schizotypy, *via* clinically significant expressions in terms of SPD diagnosis, to the most extreme psychotic expressions (18, 19, 75, 76). Moreover, schizotypy is associated with an increased risk of developing psychotic disorders in the general population; this predictive value, however, is only statistically significant over 10- to 50-year intervals (77–79) (Kwapil et al., 1998). Therefore, it appears that SPD [which is considered a clinical indicator of the latent, wider, and high order construct of schizotypy; see, e.g., Ref. (80–82)] may be more useful as a distal risk marker, detecting a more gradual progression of illness than prodrome criteria. Thus, it might fail to carry substantial clinical meaning in terms of its ability to discriminate between non-converters and converters in CHR samples. This is especially relevant among younger individuals, because more time would be required to enter the age of maximum risk for first-episode psychosis (34). Actually, among our retrieved studies, a significant psychosis-predictive role of SPD has been found in samples with a greater mean age (e.g., 23 years old) (43) and a longer follow-up period (e.g., 10 years) (37).

Overall, these results have clinical implications on current organization, validity and usefulness of UHR criteria. Along with genetic familiarity and a marked decline in psychosocial functioning, the SPD diagnosis in currently considered as an indicator of a trait vulnerability for psychosis proneness. The combination of these abovementioned risk criteria characterizes the Genetic Risk and Deterioration Syndrome (GRD), that forms a specific category of UHR syndrome [for a review, see Ref. (3)]. Despite the fact that further evidences are needed, our results on the predictive value of SPD on transition to psychosis call into question the validity of SPD as a trait risk for transition to psychotic disorders in CHR population. Interestingly, our findings are also consistent with recent meta-analytical evidence, which revealed that GRD subgroup has no higher risk of psychosis than patients that do not fulfill UHR criteria, irrespective of the length of follow-up (10).

It is important to note that the eligibility criteria of this meta-analytic review allowed us to collect studies on SPD, but not schizotypy construct dimensions. While a number of studies have focused on specific dimensions of the schizotypy construct, as well as their role in predicting psychosis transition in the CHR population, the review of such studies was not consistent with the aims of this meta-analytic review. Indeed, the schizotypy construct should be properly differentiated from SPD (80). SPD is considered a schizophrenia endophenotype on the psychosis continuum (63) and as mentioned above, a clinical indicator of the higher order latent construct of schizotypy, which can in turn be linked to a wider range of clinical and subclinical manifestations (80–82). Moreover, from the assessment standpoint, the various measures used to evaluate SPD and schizotypy are quite different. In fact, psychometric measures of schizotypy only partially overlap with SPD assessment procedures (19). For example, the negative dimensions of the Wisconsin Shizotypy Scales (physical and social anhedonia) and the interpersonal factor of the Schizotypal Personality Questionnaire (relating to social anxiety, no close friends, and flattened affect) evaluate overlapping but substantially different constructs (83). A recent review highlighted the putative predictive value of schizotypy on transition to psychosis, but it remains unclear how schizotypy features may be addressed in research on high-risk samples (19).

However, comorbid PDs diagnoses, rather than increasing the risk of conversion to psychosis, may contribute to explaining the current severe distress and disability of high-risk individuals. Currently, preventive clinical interventions usually focus on the “transition to psychosis” as the primary outcome, while the symptoms, the level of psychological functioning, and the level of distress are rarely included among treatment outcome measures. As pointed out above, it would be very useful to provide treatments for CHR individuals to promote their psychosocial well-being aside from preventing the conversion to psychotic disorders.

The comorbidity of PDs in high-risk patients might suggest putative explanations for negative outcomes of non-converters observed in longitudinal studies (84–86). Interestingly, non-converters might not have a favorable treatment outcome: one study showed that in 34–82% non-converters, attenuated psychotic symptoms persisted over 1–3 years (84); 40% had poor social or role outcomes after 3 years (86); and 75% were diagnosed with anxiety, affective, or substance use disorder after 1 year (85). It is important to consider that personality syndromes are enduring and persistent maladaptive patterns, able to incluence individual response to treatments, and, moreover, that personality changes may mediate clinically meaningful improvements in symptoms and overall psychological functioning (87, 89). PDs often require more intensive and long-term psychotherapy treatment to achieve successful outcomes (89), and their high prevalence in CHR individuals may explain the lack of evidence supporting that any specific intervention is particularly effective over others in preventing transition to psychosis (90).

#### Study Limitations

The present meta-analytic review has some limitations that should be addressed. First, the paucity of studies did not permit us to perform meta-analytic estimations of the prevalence rates for all PDs; nor did it enable us to precisely establish the psychosis-predictive role of other personality variables. Moreover, it was not possible for us to test the influence of potential moderators, such as the assessment method (self-report versus clinical interview) used to evaluate personality pathology. The impact of personality assessment procedures should be considered in future research, especially considering that self-evaluation in CHR individuals might suffer from a lack of insight and self-awareness, defensive processes, or social desirability biases [e.g., Ref. (91); see also Ref. (59)]. Second, the high variability of the reviewed studies, with respect to the assessment measures, procedures, and methods used to evaluate transition to psychosis, as well as the lengths of follow-up periods in longitudinal research designs, require conclusions to be drawn cautiously.

#### Clinical Implications

In conclusion, this meta-analytic review’s findings seem to highlight that CHR individuals may present very different personality characteristics, from the social withdrawal and affective flattening that mark schizotypal patients to the interpersonal instability and emotional dysregulation, typically shown by borderline patients. This heterogeneity could reflect the presence of distinct personality constellations that could differ in adaptive functioning, etiological variables, patterns of comorbidity, treatment response, and therapeutic interventions. Future research focused on empirically derived personality subtyping in CHR individuals and enhancing knowledge on the role that personality plays in treatment effectiveness could be promising (92). Moreover, our findings have two important clinical implications: a) treatment of UHR individuals should be integrated into interventions that are focused on maladaptive personality patterns that may moderate therapy outcomes, and b) the need to address personality features may require rethinking basic parameters of manualized treatments for at-risk mental states tested in RCTs. Surprisingly, to date, no study has addressed the effect and implication of PD diagnoses on the clinical management and treatment of CHR individuals. Psychological interventions tailored on maladaptive personality traits and disorders may provide another avenue by which to achieve symptom and functional recovery in people suffering from high-risk mental states.

## Author Contributions

Each author of the present manuscript has participated sufficiently in the work to take public responsibility for the content. TB and AT have identified, selected, and reviewed the retrieved articles. AC has performed the statistical analyses. MP, SV, and VL have supervised the work.

## Footnotes

1 This explanatory model seems particularly relevant on the basis of recent research investigating psychotic psychopathology through the lens of network theory, as applied to mental disorders (20, 21).
