# Corrigendum: Comorbid Personality Disorders in Individuals With an At-Risk Mental State for Psychosis: A Meta-Analytic Review

**DOI:** 10.3389/fpsyt.2020.00235

**Published:** 2020-05-12

**Authors:** Tommaso Boldrini, Annalisa Tanzilli, Maria Pontillo, Antonio Chirumbolo, Stefano Vicari, Vittorio Lingiardi

**Affiliations:** ^1^Department of Dynamic and Clinic Psychology, Faculty of Medicine and Psychology, Sapienza University of Rome, Rome, Italy; ^2^Department of Developmental Psychology and Socialization, University of Padova, Padova, Italy; ^3^Child and Adolescence Neuropsychiatry Unit, Department of Neuroscience, Children Hospital Bambino Gesù, Rome, Italy; ^4^Department of Psychology, Faculty of Medicine and Psychology, Sapienza University of Rome, Rome, Italy

**Keywords:** personality disorders, ultra high risk (UHR), clinical high risk (CHR), high risk (HR), early detection and prevention

In the original article, there was a mistake in **Figure 2** as published. Although the correct statistical values were reported both in the legend of **Figure 2** and in the text of the manuscript, some incorrect values were reported in **Figure 2** due to a copy and paste error. In addition, the wrong years were listed is some of the study names. The corrected **Figure 2** appears below.

**Figure 2:**
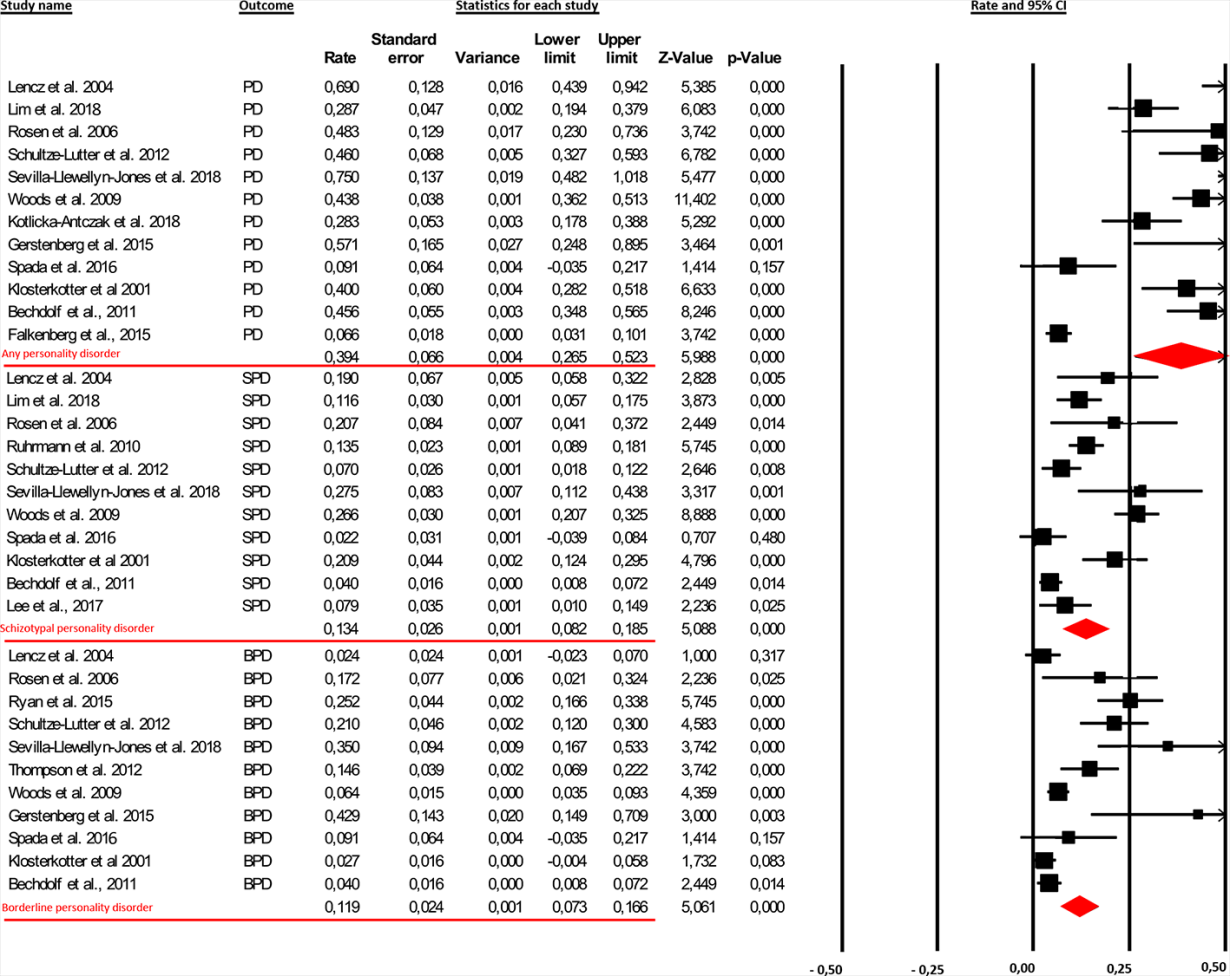
The findings showed that the prevalence rate of comorbid personality diagnoses in clinical-high-risk (CHR) patients was 39.4% [95% Cl (26.5%–52.3%)]. More specifically, 13.4% [95% Cl (8.2%–18.5%)] and 11.9% [95% Cl (0.73%–16.6%)] of this clinical population presented with the schizotypal personality disorder (SPD) and borderline personality disorder (BPD), respectively.

The authors apologize for these errors and state that this does not change the scientific conclusions of the article in any way. The original article has been updated.

