# Supplementary material for: Comorbid Personality Disorders in Individuals With an At-Risk Mental State for Psychosis: A Meta-Analytic Review
Source: Front Psychiatry. 2019 Jul 5;10:429. doi: 10.3389/fpsyt.2019.00429 (PMC6625011; doi:10.3389/fpsyt.2019.00429)
Supplement: Supplementary file 1 [file Table_1.doc]

**Supplementary** material. Quality assessment for the studies included in the meta-analytic review

| Study | Selection | Comparability | Exposure | NOS stars |
| --- | --- | --- | --- | --- |
| Bechdolf et al. 2011 | ⋆ ⋆ ⋆ ⋆ | ⋆ ⋆ | ⋆ ⋆ ⋆ | 9 |
| Cannon et al. 2008 | ⋆ ⋆ ⋆ ⋆ | ⋆ ⋆ | ⋆ ⋆ ⋆ | 9 |
| Falkenberg et al. 2015 | ⋆ ⋆ ⋆ ⋆ | ⋆ ⋆ | ⋆ ⋆ | 8 |
| Gerstenberg et al. 2015 | ⋆ ⋆ ⋆ | ⋆ | ⋆ ⋆ | 6 |
| Klosterkötter et al. 2001 | ⋆ ⋆ ⋆ ⋆ | ⋆ ⋆ | ⋆ ⋆ ⋆ | 9 |
| Kotlicka-Antczak et al. 2018 | ⋆ ⋆ ⋆ ⋆ | ⋆ ⋆ | ⋆ ⋆ | 8 |
| Lee et al. 2017 | ⋆ ⋆ | ⋆ | ⋆ ⋆ | 5 |
| Lencz et al. 2004 | ⋆ ⋆ ⋆ | ⋆ | ⋆ ⋆ | 6 |
| Lim et al. 2018 | ⋆ ⋆ ⋆ ⋆ | ⋆ ⋆ | ⋆ ⋆ | 8 |
| Rosen et al. 2006 | ⋆ ⋆ ⋆ | ⋆ | ⋆ ⋆ | 6 |
| Ruhrmann et al. 2010 | ⋆ ⋆ ⋆ ⋆ | ⋆ | ⋆ ⋆ | 7 |
| Ryan et al. 2015 | ⋆ ⋆ ⋆ ⋆ | ⋆ ⋆ | ⋆ ⋆ | 8 |
| Schultze-Lutter et al. 2012 | ⋆ ⋆ ⋆ ⋆ | ⋆ ⋆ | ⋆ ⋆ | 8 |
| Sevilla-Llewellyn-Jones et al. 2018 | ⋆ ⋆ ⋆ | ⋆ | ⋆ ⋆ | 7 |
| Spada et al. 2016 | ⋆ ⋆ | ⋆ | ⋆ ⋆ | 5 |
| Thompson et al. 2012 | ⋆ ⋆ ⋆ ⋆ | ⋆ ⋆ | ⋆ ⋆ | 8 |
| Woods et al. 2009 | ⋆ ⋆ ⋆ ⋆ | ⋆ | ⋆ ⋆ | 7 |

Note: NOS, Newcastle-Ottawa Scale

Subsections: Selection (max 4 stars); Comparability (on the basis of design - max 2 stars); Exposure (max 3 stars)
